# Alpha-synuclein null mutation exacerbates the phenotype of a model of Menkes disease in female mice

**DOI:** 10.3389/fnins.2025.1613171

**Published:** 2025-07-03

**Authors:** MegAnne Casey, Dan Zou, Renee A. Reijo Pera, Tiffany Hensley-McBain, Deborah E. Cabin

**Affiliations:** McLaughlin Research Institute, Weissman Hood Institute at Touro University, Great Falls, MT, United States

**Keywords:** Parkinson’s disease, Menkes disease, *Atp7a*, neurodegeneration, ENU mutagenesis

## Abstract

Human *SNCA*, which encodes a-synuclein protein (*SNCA*), was the first gene linked to familial Parkinson’s disease (PD). Since the discovery of the genetic link of *SNCA* to Parkinson’s nearly three decades ago, many studies have investigated the normal function of *SNCA* protein. However, understanding of the normal function of *SNCA* is complicated by the lack of a reliable mammalian model of PD; indeed, mice with homozygous null mutations in the *Snca* gene live a normal lifespan and have only subtle synaptic deficits. Here, we report the first genetic modifier (a sensitized mutation) of a murine *Snca* null mutation, namely the *ATPase copper transporting alpha (Atp7a),* an X-linked gene that escapes inactivation in both mice and humans. In humans, mutations in *Atp7a* are linked to Menkes disease, a disease with pleiotropic and severe neurological phenotypes. *Atp7a* encodes a copper transporter that supplies the copper co-factor to enzymes that pass through the ER-Golgi network; under some conditions, *Atp7a* protein may also act to increase copper flux across the cell membrane. Male mice that carry a mutation in *Atp7a* die within 3 weeks of age regardless of *Snca* genotype. In contrast, female mice that carry the *Atp7a* mutation, on an *Snca* null background, die earlier (prior to 35 days) at a significantly higher rate than those that carry the *Atp7a* mutation on a wildtype *Snca* background. Thus, *Snca* null mutations sensitize female mice to mutations in *Atp7a,* suggesting that *Snca* protein may have a protective effect in females, perhaps in neurons, given the co-expression patterns. This study adds to the growing literature suggesting that alterations in a-synuclein structure and/or quantity may manifest in neurological differences in males and females including phenotypes of developmental delays, seizures, muscle weakness and cognitive function.

## Introduction

Parkinson’s disease (PD) is the second most common neurodegenerative disease in humans with Alzheimer’s disease being the most common (Kochanek et al., 2023). While the majority of cases are late-onset and sporadic, genetic forms of PD have also been identified (Schneider and Alcalay, 2020; Over et al., 2021; Xia et al., 2022). *Alpha-synuclein* (*SNCA*) was the first gene identified as causing a genetic form of PD (Polymeropoulos et al., 1997). *SNCA* protein was subsequently shown to be a major component of Lewy bodies (Spillantini et al., 1997) with intracellular inclusions commonly observed in postmortem midbrain tissue in conjunction with PD. The basis of *SNCA* toxicity in PD is not well understood; human mutations that have been identified include both loss-of-function (rare missense mutations) and gain-of-function (numerical variant) mutations (Farrer et al., 1998; Singleton et al., 2003; Zarranz et al., 2004; Guzzo et al., 2021). Potential mechanisms for toxicity have centered on the concept of accumulation of toxic wildtype or mutant protein species (Volles and Lansbury, 2003); however, the mechanism of toxicity and identity of the toxic form (s) are not known and continue to be a subject of debate (Guzzo et al., 2021; Lassen et al., 2022; Virdi et al., 2022; Yoo et al., 2022; Bagree et al., 2023). Clearly, however, *SNCA* is able to form a variety of oligomeric structures, including the fibrils found in Lewy bodies.

Numerous studies have reported on diverse functions of *SNCA* in different cellular compartments including the synapse, mitochondria, nucleus, endoplasmic reticulum, and cytoplasm. Functional analysis is complicated by the fact that mice that lack *Snca* protein are overall healthy and live a normal lifespan with only subtle synaptic phenotypes (Abeliovich et al., 2000; Cabin et al., 2002; Dauer et al., 2002). Over-expression of wild type human *SNCA* in mice has been shown to rescue the phenotypes of mice lacking the synaptic chaperone cysteine string protein suggesting a direct or indirect interaction between these proteins (Chandra et al., 2005). In other studies, *Snca* has also been shown to enhance SNARE assembly at the synapses in mice and additional mechanisms of toxicity, including inhibition of ER to Golgi trafficking, have also been proposed (Cooper et al., 2006; Burré et al., 2010; Thayanidhi et al., 2010; Burré et al., 2018; Guzzo et al., 2021; Lassen et al., 2022; Virdi et al., 2022; Yoo et al., 2022; Bagree et al., 2023). Other functions of *SNCA* include modulation of mitochondrial structure and function (Park et al., 2020; Choudhury et al., 2022), regulation of gene expression (Schaser et al., 2019; Chen et al., 2020; Somayaji et al., 2021; Koss et al., 2022), epigenetic modification (Pavlou et al., 2016), nuclear transport (Chen et al., 2020; Koss et al., 2022), neuronal survival (Benskey et al., 2016), cytoskeletal stabilization (Carnwath et al., 2018) and even DNA repair (Schaser et al., 2019).

In this study, we probed the function of *Snca* further by focusing on modifiers of the phenotypes associated with *Snca* null alleles in mice. For this purpose, we used a sensitized mutagenesis screen in mice as previously described (Justice et al., 1999; Soewarto et al., 2003). The use of modifier screening to identify sensitizing mutation(s) has the advantage of being independent of *a priori* assumptions such as *Snca* function, cellular and subcellular localization, and physical properties, relative to other common methods of identifying interactions (Goers et al., 2003; Soewarto et al., 2003). Thus, the goal here was to identify mice with ENU-induced mutations that result in neurological phenotypes that are more severe in the absence of *Snca* gene function than in the presence of *Snca* gene function. These ENU-induced mutations could provide information on genetic pathways in which the *Snca* gene functions.

## Results

**Figure 1:**
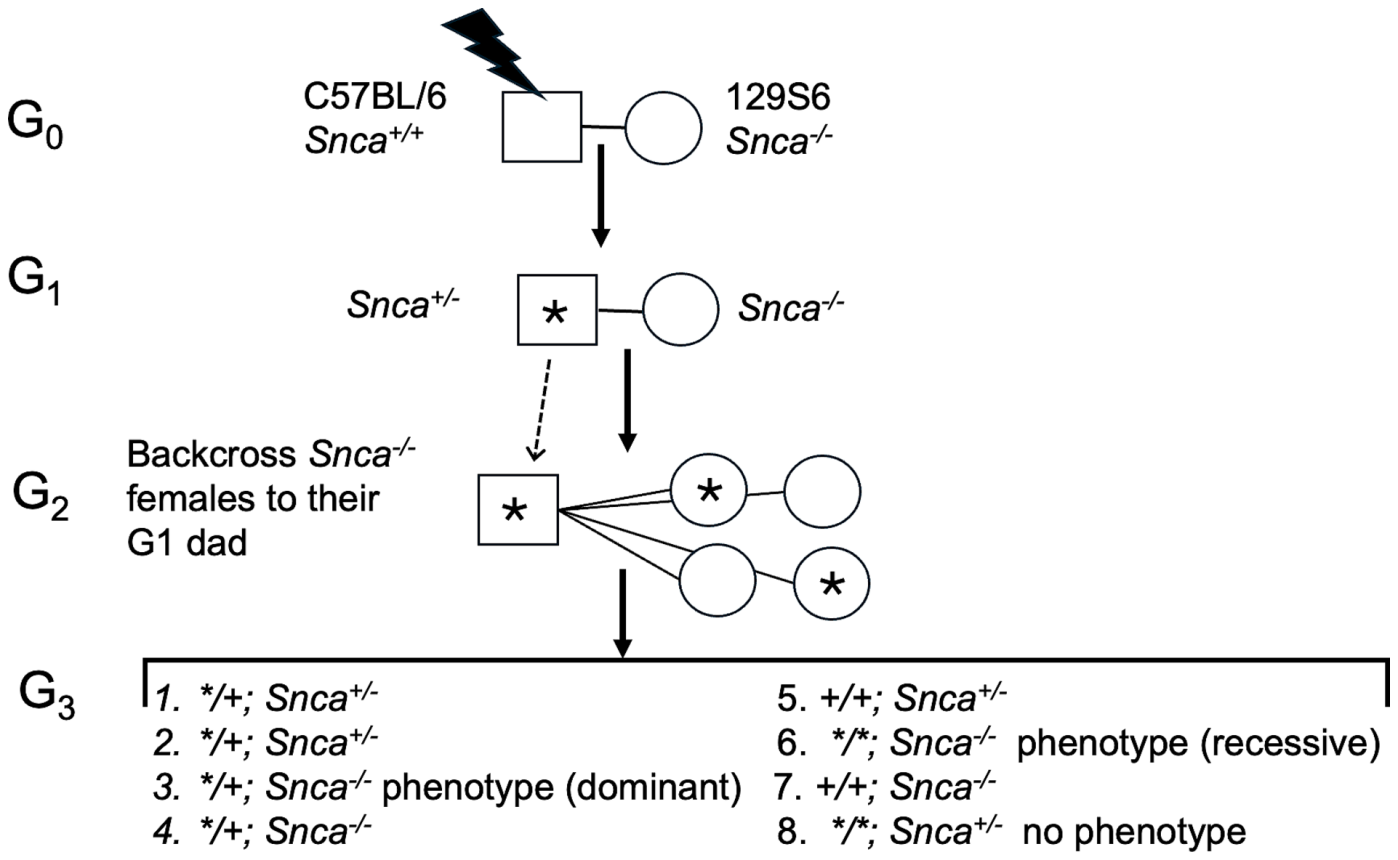
Diagram of ENU (N-ethyl-N-nitrosourea) screen and breeding scheme to identify modifying alleles. “*” Denotes ENU-induced mutation. Male mice were mutagenized with ENU, then bred to females homozygous null for *Snca;* offspring were outcrossed to uncover recessive mutations that are more severe in the absence of *Snca* gene function than on a wild type background (“sensitized”). Approximately 30–40 G3 offspring per line were assessed for neurological phenotypes with 125 pedigrees screened through the G3 stage.

Our ENU (N-ethyl-N-nitrosourea) screen followed a standard protocol (Figure 1): C57BL/6 male mice were mutagenized with ENU, then bred to females homozygous null for *Snca,* and offspring were outcrossed in a breeding scheme designed to uncover recessive mutations that are more severe in the absence of *Snca* gene function than on a wild type background (“sensitized”). Approximately 30–40 G3 offspring per line were assessed for neurological phenotypes with 125 pedigrees screened through the G3 stage. Our screen for neurological phenotypes was based on the modified SHIRPA (SmithKline Beecham, Harwell, Imperial College, Royal London Hospital, Phenotype Assessment) protocol established in a large ENU-mutagenesis screen over two decades ago (Nolan et al., 2000; Lalonde et al., 2021). The SHIRPA protocol is a battery of tests comprising 42 measurements of motor activity, coordination, postural control, muscle tone, autonomic functions, and emotional reactivity, as well as reflexes dependent on visual, auditory, and tactile modalities (Lalonde et al., 2021). Of 125 pedigree lines examined, 7 were followed further based on the SHIRPA analyses that revealed: (1) Extreme reaction to clicker with clasping of forepaws, (2) poor performance on bar, (3) circling behavior, (4) over reaction to clicker, hyperactivity and odd gait, (5) premature dropping from bar, (6) hard landing, bouncing and little reaction in clicker testing, and (7) poor performance on bar with clasping of hind paws.

**Figure 2:**
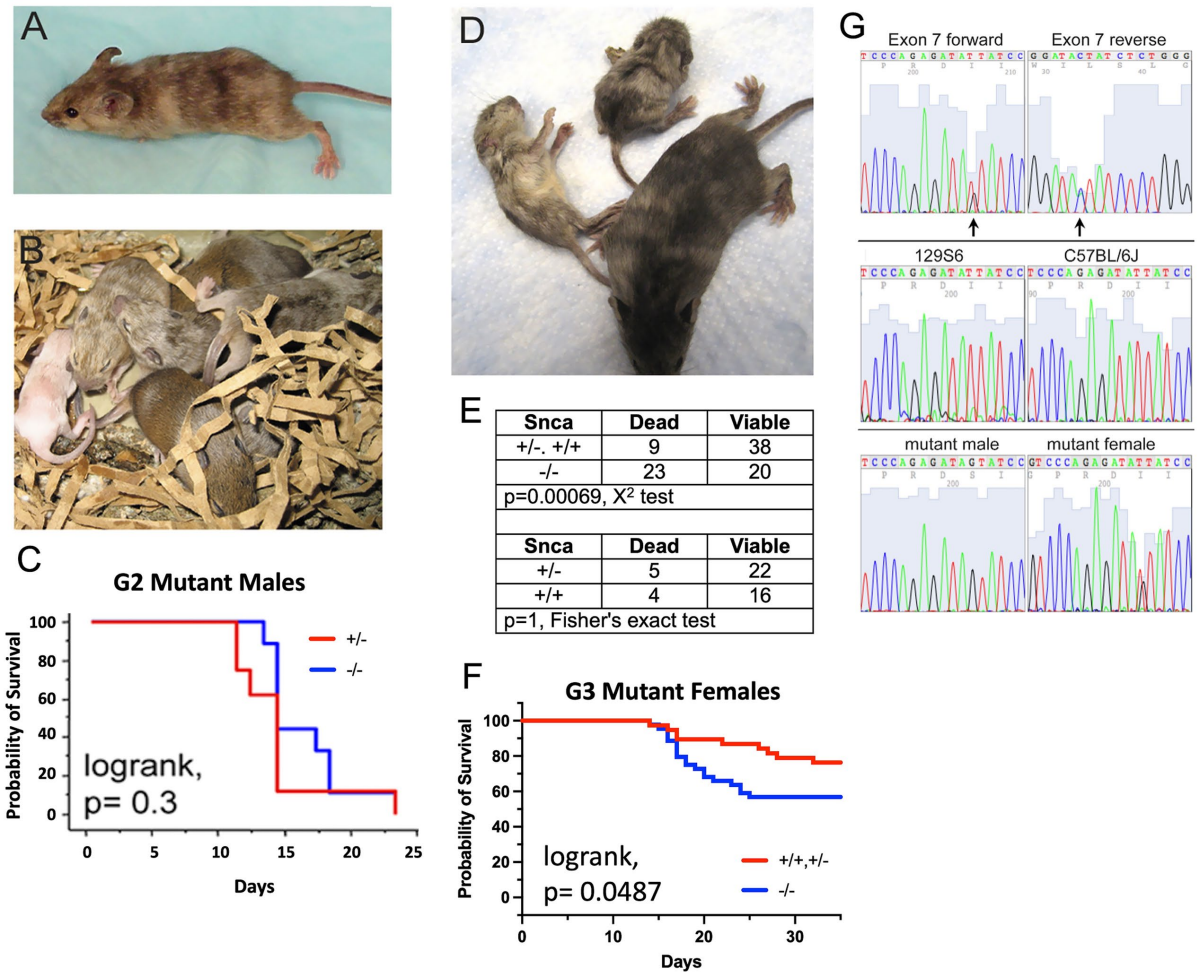
C57BL/6J males were treated with ENU, allowed to recover fertility, and mated to *Snca* null females on a 129S6 background. G1 males were used in further breeding to uncover sensitized recessive mutations. The mouse coat color mutation is X-linked. **(A)** One of 2 original G1 females identified as carrying a coat color mutation. **(B)** Litter of G2 pups showing a white male, two females with mottled coat color and agouti littermates. **(C)** Kaplan-Meyer lifespan analysis shows that lack of *Snca* does not affect lifespan of coat color mutant males (*n* = 8 per group). **(D)** *Snca* null, coat color mutant female G3 littermates at 4 weeks of age, showing one viable and two dying animals. **(E)** Table showing the frequency of G3 female offspring, carrying the coat color mutation. Dying within 35 days of birth is significantly increased on a *Snca* null background relative to homozygous or heterozygous wildtype. **(F)** Kaplan-Meyer lifespan analysis shows that lack of *Snca* significantly affects lifespan of coat color mutant females (*n* = 37 for homozygous/heterozygous wildtype and *n* = 43 for *Snca* null). **(G)** Identification of a mutation in *Atp7a* that segregates with the coat color phenotype; (top panel) forward and reverse exon 7 sequence from a coat color mutant female identifies coding sequence nucleotide 1951 as heterozygous (arrows); (middle) exon 7 forward sequence from females of the two strains used in the ENU mutagenesis procedure, 129S6 and C57BL/6J; (bottom) exon 7 forward sequence from an affected male is hemizygous for the mutant nucleotide, and an additional affected female is heterozygous.

In the second pedigree of the screen, two pheno-deviant G1 females were identified by their patchy coat color (Figure 2A). The patchiness suggested X-linkage of the underlying mutation. We surmised that a good candidate, X-linked gene was *Atp7a*, a copper transporter implicated in neurodegeneration. Indeed, mutations in *ATP7a* are known to cause X-linked Menkes disease in humans, a disease with a severe neurological component. The G1 female pheno-deviants were heterozygous for the *Snca* null allele and thus, were bred to *Snca* null mice to determine if the mutation was indeed X-linked, and whether lack of *Snca* affected the severity of the phenotype. A litter is shown in Figure 2B; the coat color of mutant males was all white while affected females had a patchy coat color, further confirming X-linkage. We observed that all coat color mutant males died <25 days; further analysis of life span also indicated that there was no difference in lifespan between *Snca* null and *Snca* heterozygotes of the mutant males (Figure 2C). In contrast to observations with male coat color mutant mice, female coat color mutants that were also homozygous *Snca* null mutants demonstrated increased early death (<35 days) relative to mice heterozygous or wildtype for *Snca*, though statistical significance was not reached with the low number of affected female offspring from the two G1 females (data not shown). Subsequently, G2 coat color mutant females were bred to both *Snca* nulls and to wildtype controls (on the 129S6 genetic background) to obtain greater numbers for comparisons. An example of viable and inviable *Snca* null coat color mutant female littermates is shown in Figure 2D. Further analysis indicated that the G3 offspring demonstrated a significantly higher rate of early death (<35 days) in the *Snca* null coat color females vs. *Snca* heterozygotes or wildtype (Figures 2E,F). Thus, we confirmed that the coat color mutation is sensitized by the null mutation in *Snca*.

As *Atp7a* was the best candidate gene, a mutant female’s *Atp7a* coding region DNA was sequenced and a mutation was identified at nucleotide position1951 (NM_001109757) in exon 7 (Figure 2G), which was heterozygous for G and T. The G changes the amino acid at position 610 from an isoleucine to a serine (NP_001103227; this corresponds to human amino acid 618, NP_000043). More than 400 nonsense, missense and insertions/deletions have been identified in the human *ATP7a* gene; those associated with Menkes disease are within the protein coding sequence and map to the copper associated domains, ATPase or transmembrane domains in particular (Mhaske et al., 2020). In the two parental strains used in the mutagenesis protocol, 129S6 and C57BL6/J, the nucleotide at position 1951 is a T (Figure 2G). The change to a G at this position segregated with the coat color phenotype; sequences from an affected male and a second affected female are shown in Figure 2G. Amino acid 610 lies between the 6th of six metal binding domains in *Atp7a* and the first transmembrane domain, a position of functional importance based on previous studies (Lutsenko et al., 2007).

**Figure 3:**
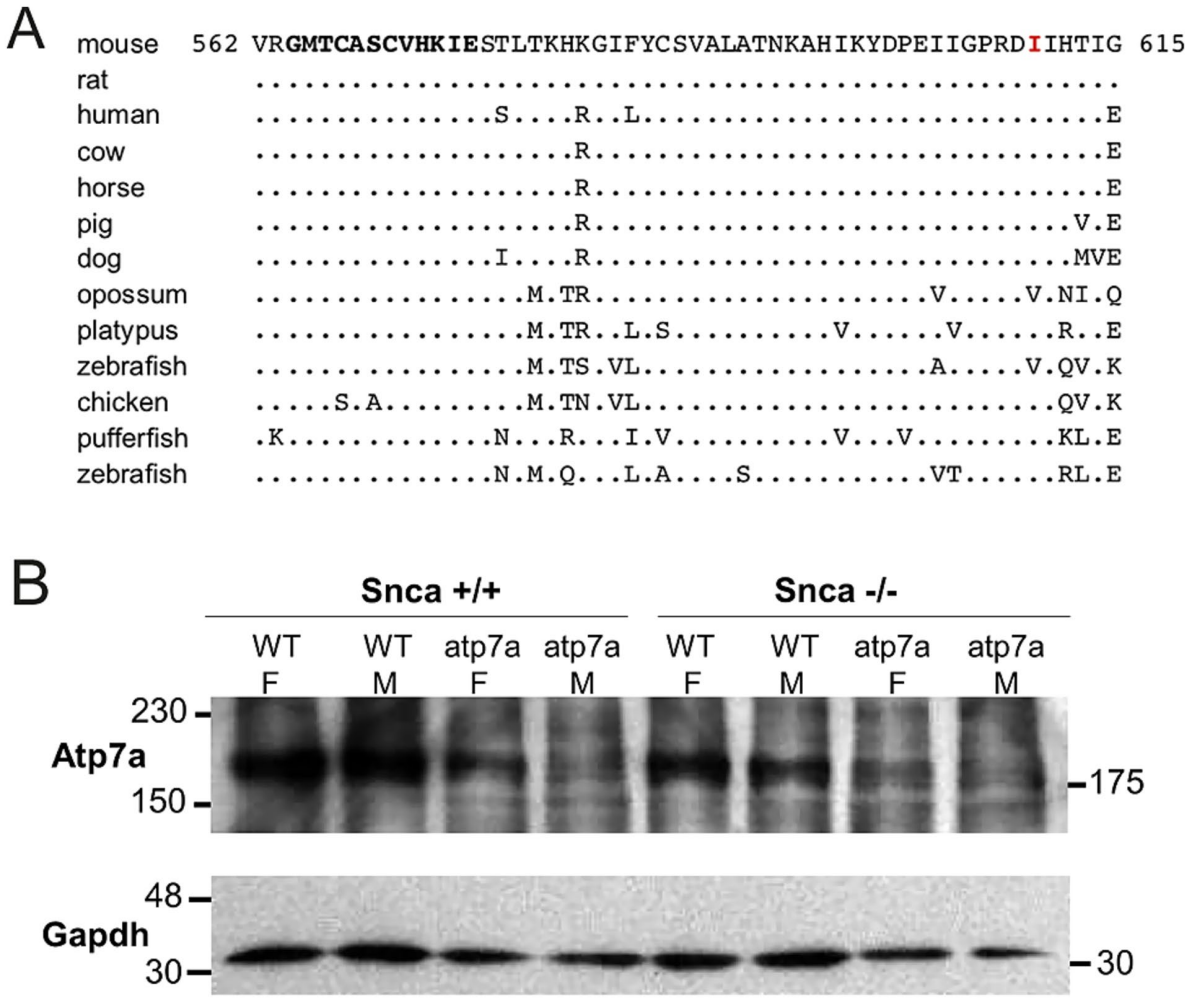
Isoleucine 610 is highly conserved, but *Atp7a* protein is still produced in the I610S mutant mice. **(A)** Alignment of exon 7 amino acid sequences. The mutated I610 is indicated in red, and the sixth *Atp7a* copper-binding domain is in bold towards the N-terminus. The consensus copper-binding domain is GMT/HCxxCxxxIE. **(B)** Western blot of total brain lysates from P8 mice, with Gapdh protein as a loading control.

A multi-species protein alignment of exon 7 is shown in Figure 3A. The isoleucine at position 610 is well conserved, though a conservative substitution of a valine is found in both the opossum and zebrafish. Numerous *Atp7a* alleles are known in mice, and most cause affected males to die *in utero* (Silvers, 1979). The observation that males carrying the I610S mutation do not die until 2 to 3 weeks postnatally suggests that this new allele may be hypomorphic resulting in only partial loss-of-function. Northern blot analysis indicated similar amounts of *Atp7a* mRNA in both male and female mutant brains (data not shown). Similarly, Western blot analysis with total brain lysates from postnatal day 8 pups showed that *Atp7a* protein is made in all mutant animals, though at a lower level than in wildtype animals. Further, the absence of *Snca* protein did not alter *Atp7a* levels (Figure 3B). Similar results were observed in postnatal day 14 animals (data not shown).

**Figure 4:**
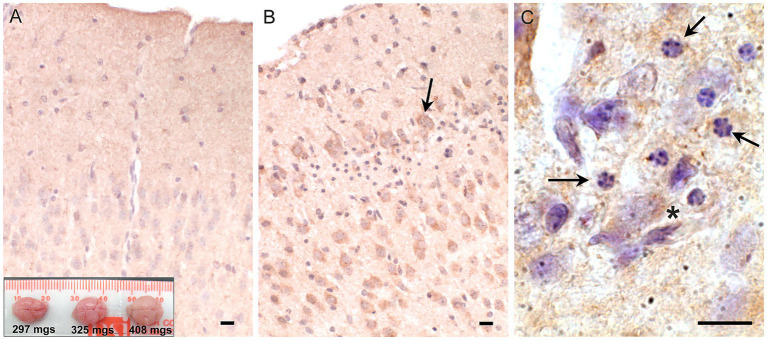
Apoptosis in cerebral cortex of inviable *Atp7a^I610S^ Snca* null female mice. Cleaved caspase-3 immunohistochemistry. **(A)** Cingulate cortex from viable *Snca−/−; Atp7a^I610S^* female shown in Figure 1A; inset; brains from the three animals in Figure 1A, viable on the right. **(B)** Cingulate cortex from inviable *Snca−/−; Atp7a^I610S^* female; arrow indicates cleaved-caspase-3 positive neuron above layer of pyknotic nuclei. **(C)** Higher magnification of pyknotic nuclei (arrows) and cleaved caspase-3 positive neuron (asterisk). Scale bars = 0.05 mm.

*Snca* is most abundant in neurons and thus we reasoned, it may have a neural protective effect in females. Aneurysms caused by faulty collagen maturation were a frequent cause of death in *Atp7a* male mutant mice that survive postnatally, and *Snca* clearly did not appear to impact death or survival. In females, random X-inactivation of the mutant chromosome could circumvent phenotypic problems associated with collagen dysfunction at least in part. We hypothesized that while random X-inactivation would also occur in neurons, some neuronal populations might be more vulnerable to the effects of mutant *Atp7a* in the absence of *Snca*. Thus, to test our hypothesis, we compared brain samples from viable and dying *Snca*−/−; *Atp7a^I610S^* female littermates via an antibody against cleaved caspase-3 to identify apoptotic cells (Figure 4). The cerebral cortex appeared to be smaller relative to the cerebellum in brains from dying mice (Figure 4A, inset), and patches of cortical neurons positive for cleaved caspase-3 were observed only in dying but not in viable.

*Snca*−/−; *Atp7a^I610S^* female littermates (Figures 4A,B). In addition to cleaved caspase-3 positivity as an indicator of apoptosis, haematoxylin counterstain also demonstrated patches of large numbers of smaller pyknotic nuclei in cortical layers II/III in dying but not in viable females (Figures 4B,C).

**Figure 5:**
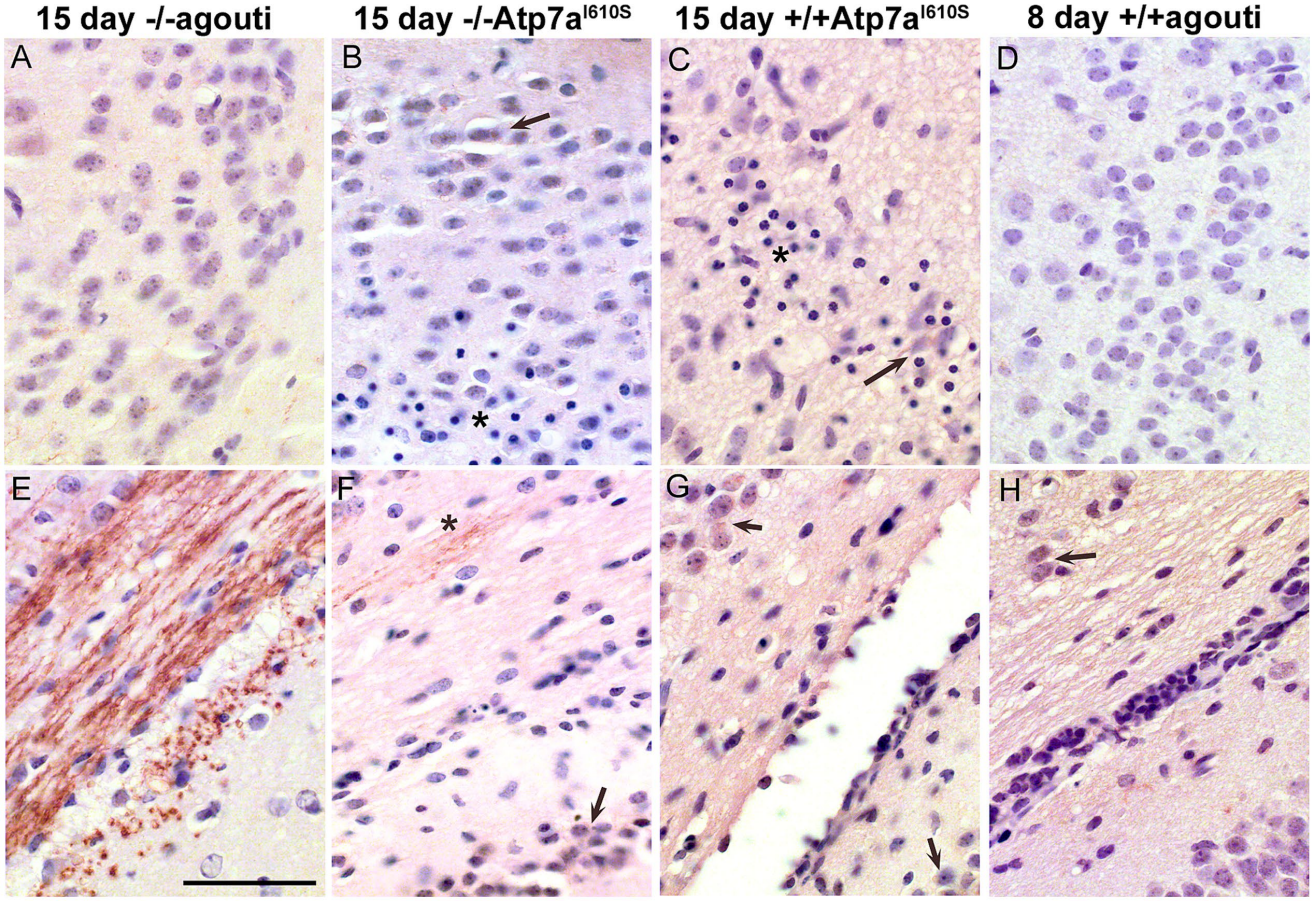
*Atp7a* in male control and mutant cerebral cortex and corpus callosum. *Atp7a* immunohistochemistry on brains of *Snca−/−; Atp7a^I610S^*, *Snca+/+; Atp7a^I610S^*, and control animals at 15 days of age, as well as an 8 day control to match brain size of the 15 day old mutants. **(A–D)** Cerebral cortex. In mutant animals, **(B,C)** arrows indicate cell body localization of *Atp7a*; asterisks indicate the regions with pyknotic nuclei. **(E–H)** Corpus callosum. **(E)** At 15 days, *Atp7a* is strongly expressed in wild type. **(F)** Asterisk indicates minor neuritic-like *Atp7a* localization. **(F–H)** Arrows indicate cell body localization of *Atp7a*. Scale bar for all panels = 0.05 mm.

*Atp7a* immunohistochemistry was performed on brains from male mice in which only one allele of *Atp7a* was expressed. Sections of postnatal day fifteen brains from *Atp7a^I610S^* males were reduced in size relative to age-matched controls, so sections of postnatal day eight control brains of similar size were also analyzed by immunohistochemistry. Greater numbers of pyknotic nuclei were detected in cerebral cortex, mostly in layers II/III, in *Atp7a* mutant brains relative to controls (Figures 5A–D). Immunohistochemical staining of *Atp7a* was strong in the corpus callosum in fifteen-day old control animals (Figure 5E). Staining was weaker in 15 day mutant males (Figures 5F,G), but the 15 day *Snca*+/+; *Atp7a^I610S^* pattern more closely resembled that of the 8 day control (Figure 5H) than does the 15 day *Snca*−/−; *Atp7a^I610S^* male. Analysis of *Snca*−/−; *Atp7a^I610S^* brain samples suggested a delay in axonal localization of *Atp7a*, while the *Snca*+/+; *Atp7a^I610S^* brain samples had more diffuse staining similar to the 8 day controls. *Atp7a* staining of cell bodies is more apparent in the fifteen-day old mutants and the eight day controls than in the fifteen day control brain.

**Figure 6:**
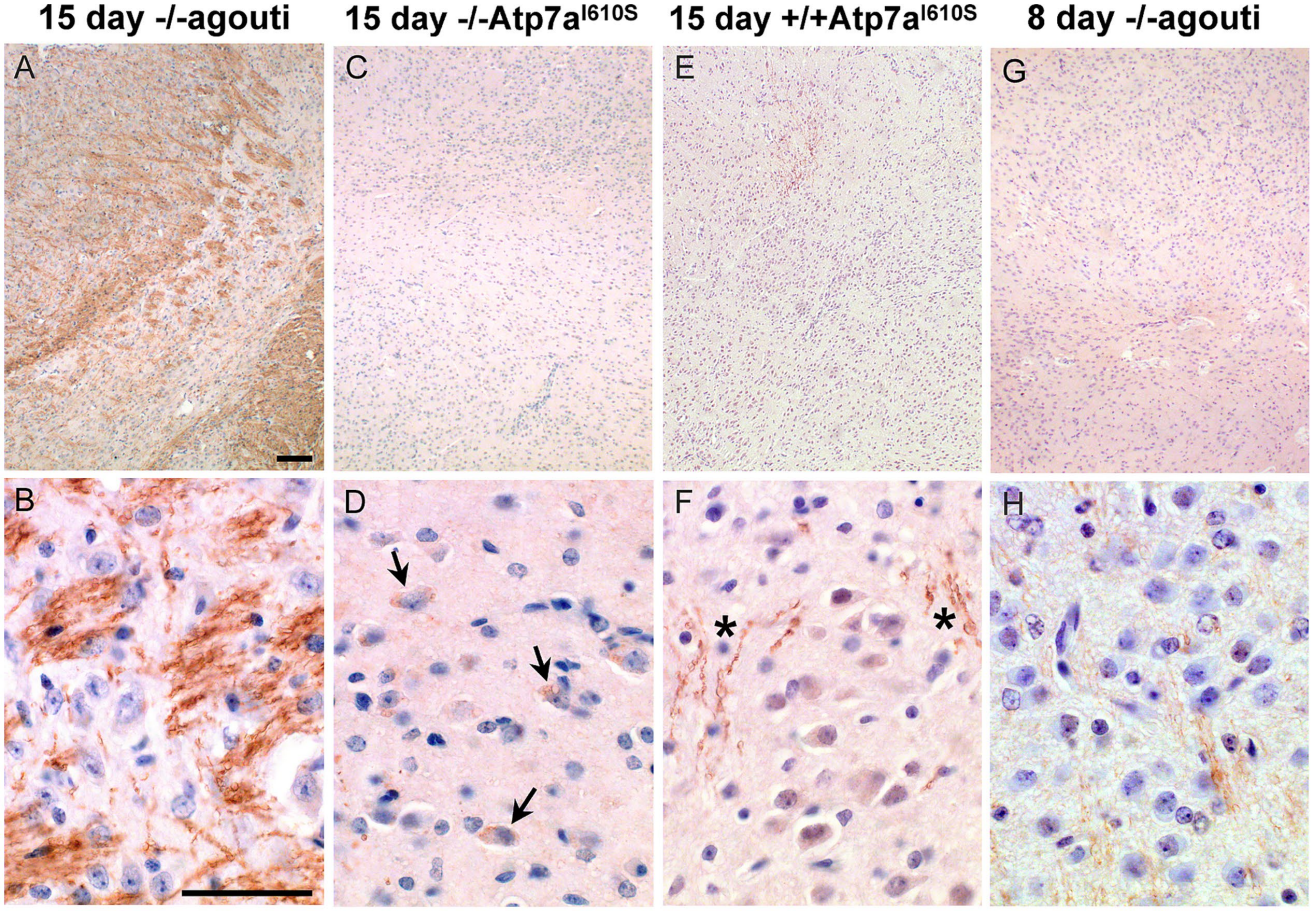
*Atp7a* in developing striatum. *Atp7a* immunohistochemistry on striatum from male mice. Top panels, low magnification, scale bar = 0.1 mm; bottom panels high magnification, scale bar = 0.05 mm. **(A,B)** Wild type control shows robust *Atp7a* expression in striatum. **(C–F)** 15 day mutant males lag in development of the striatum and appear similar to the 8 day control in panels **(G,H)**. Arrows in **(D)** indicate cell body localization of *Atp7a* in the *Snca−/−; Atp7a^I610S^* animal; asterisks in **(F)** show some neuritic staining in the *Snca+/+; Atp7a^I610S^* animal.

Strong *Atp7a* staining was observed in the developing striatum in the fifteen-day control brain (Figures 6A,B), in a neuritic pattern. Figure 6A shows an *Snca* null brain with staining similar to *Snca* wild type brain samples (data not shown). The defined pattern of *Atp7a* staining in axonal sub-compartments of the striatum was not established in the corresponding region from fifteen day mutant and eight day old control brains (Figures 6C–H). In the *Snca*−/−; *Atp7a^I610S^* brain, *Atp7a* was localized primarily to cell bodies (Figure 6D). However, in the presence of *Snca*, some faint neuritic staining was apparent in fifteen-day mutant brain samples (Figures 6E,F), although cell body staining was more prominent than in the fifteen-day control. In the absence of *Snca* (on an *Snca* null background), faint staining of wild type *Atp7a* was detected in a neuritic pattern in eight-day old brain. Thus, *Snca* may aid in the proper localization of the mutant form of *Atp7a*, but is not required by the wild type protein.

**Figure 7:**
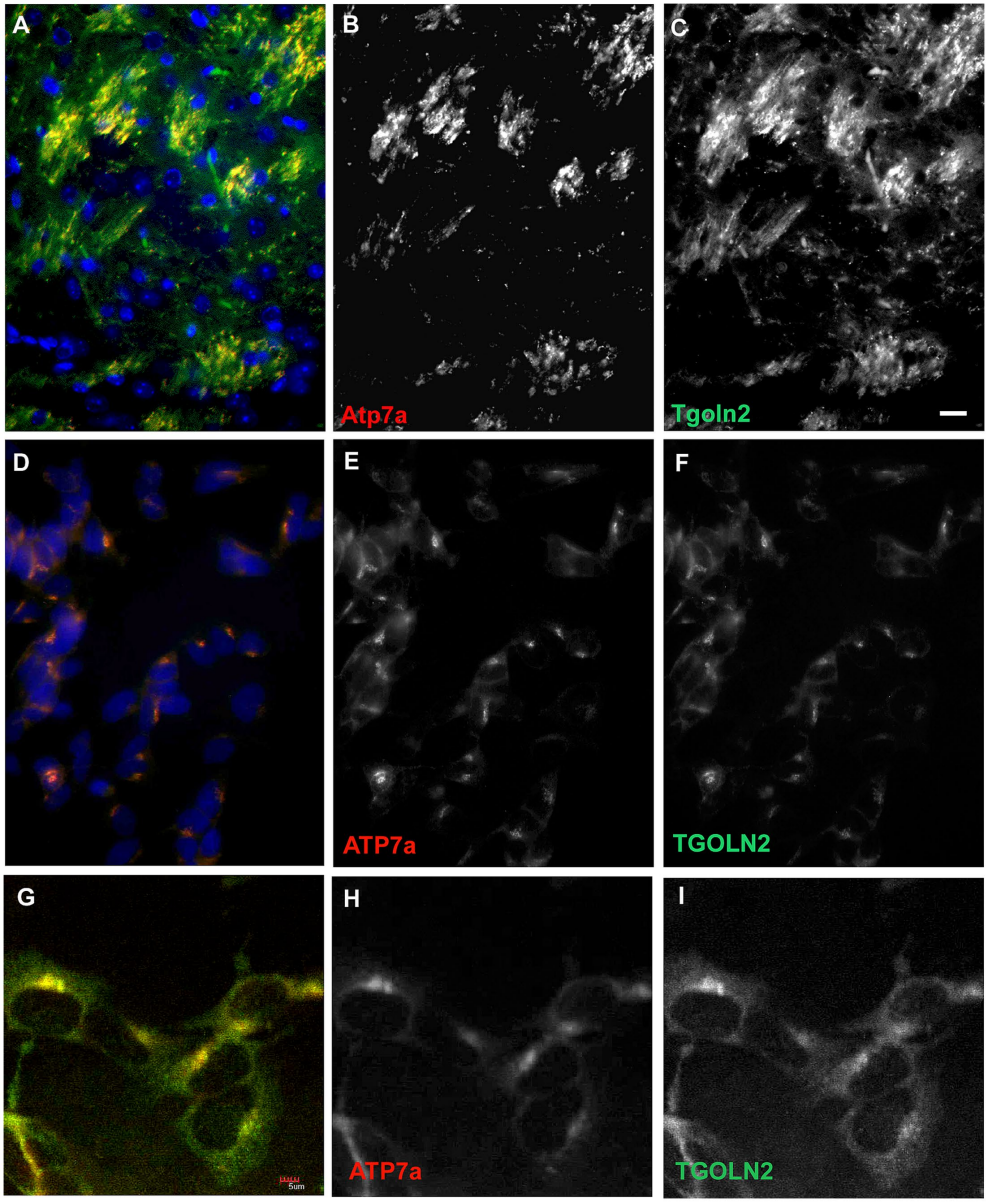
A trans-Golgi marker protein, *Tgoln2*, co-localizes with *Atp7a* in mouse brain and in SH-SY5Y cells. **(A–C)** Striatum in 22 day old wild type mouse. **(A)** Merge, with DAPI in blue. **(B)** *Atp7a*, **(C)**, *Tgoln2*. **(D–F)** SH-SY5Y cells express *ATP7a* and *TGOLN2* in the same peri-nuclear compartment. **(D)** Merge, with DAPI in blue. **(E)** *ATP7a*. **(F)** *TGOLN2*. Scale bar for **(A–F)** = 0.05 mm. **(G–I)** Single slice confocal image of SH-SY5Y cells. **(G)** Merge. **(H)** *ATP7a*. **(I)** *TGOLN2*. Scale bar for **(G–I)** = 0.005 mm.

Neuritic localization of *Atp7a* was unexpected. However, *Atp7a* has been reported to play a role in axonal targeting and synaptogenesis in olfactory bulb (El Meskini et al., 2007; Niciu et al., 2007). We used immunohistochemistry with trans-Golgi marker, *Tgoln2*, in double labeling experiments to determine if neuritic *Atp7a* was associated with the trans-Golgi compartment. In wildtype striatum from 22-day old mice, *Atp7a* and *Tgoln2* showed a similar pattern of localization (Figures 7A–C). *Atp7a* signal was almost completely co-localized with the *Tgoln2* signal (Figure 7A). Similar results were seen in axonal tracts of the wild type corpus callosum connecting the hemispheres (not shown). In *Atp7a^I610S^* brains, the *Atp7a* signal was faint in corpus callosum, but overlapped with that of *Tgoln2* (not shown). SH-SY5Y cells were then tested to confirm that the antibodies used were indeed targeting proteins of the proper compartment. In these undifferentiated cells, the antibodies against *ATP7a* and *TGOLN2* both identify a perinuclear region that is consistent with the Golgi compartment (Figures 7D–F). Higher magnification confocal imaging indicated that the two proteins are found in the same perinuclear compartment (Figures 7G–I).

## Discussion

*Snca* protein is present in multiple cellular compartments and ubiquitously expressed, though it is most abundant in the nervous system where it may comprise as much as 1% of the total protein in both humans and mice (Vivacqua et al., 2011; Wang et al., 2024). In this work, we demonstrate that a mutation in the *Atp7a* gene produces a more severe phenotype on a null *Snca* background, relative to homozygous or heterozygous wildtype, in female mice only (*p* = 0.00069). The *Atp7a* protein is a well-characterized protein that is required for copper transport, for loading copper onto copper-dependent enzymes, removing excess copper from the cell to maintain homeostasis and for facilitating ER to Golgi transport processes (Cooper et al., 2006; Thayanidhi et al., 2010; Telianidis et al., 2013). Neurodegeneration has been linked to metal ion transport across disease types including Menkes disease which is directly linked to mutations in *Atp7a*; other neurodegenerative diseases including, Parkinson’s disease, Alzheimer’s disease, frontal temporal dementia and amyotrophic lateral sclerosis (ALS) (Gale and Aizenman, 2024). This work uncovered a genetically link between the *Snca* and *Atp7a* gene functions, major proteins of Parkinson’s disease and Menkes disease, respectively, via a modifier screen.

The first phenodeviant mice identified in our screen were two female littermates identified by a patchy coat color phenotype. The patchy coat color suggested X-linkage, and we surmised that the best X-linked candidate gene might be *Atp7a*, a trans-Golgi copper transporter mutated in Menkes disease in humans (OMIM 309400). This disease affects many organ systems, including the nervous system (see review, Tümer and Møller, 2010). All enzymes that pass through the ER-Golgi pathway and require copper as a co-factor are impaired in Menkes disease. These include tyrosinase, causing pigmentation defects, and lysyl oxidase, which is required for collagen maturation. Two neuronal enzymes that require copper are dopamine *β*-hydroxylase, which converts dopamine to norepinephrine, and peptidyglycine α-amidating monooxygenase, required for neuropeptide amidation, a post-translational modification that many neuropeptides require for full activity (Lutsenko et al., 2010).

We decided to test whether lack of *Snca* had any effect on the severity of the phenotypes in our mouse coat color mutants for several reasons. First, Menkes disease has a severe neurological component that can progress to complete loss of cerebral brain function (Aaltio et al., 2024). Second, like *Atp7a*, *SNCA* has also been shown, in some studies, to affect ER-Golgi trafficking in yeast and mammalian cells (Thayanidhi et al., 2010); moreover, occasional reports have also suggested that *SNCA* may bind copper (Brown, 2007; Davies et al., 2011). Finally, it has been shown that the incidence of PD is higher amongst melanoma patients, linking *SNCA* to pigmentation (see review, Pan et al., 2011). We demonstrate here that lack of *Snca* significantly increased incidence of early death in female coat color mutant mice carrying a missense mutation in *Atp7a.* Many studies have sought to identify direct protein–protein interactions that regulate *SNCA* pathology; proteins that may directly interact are many including Tau, Ab, TDP-43, PrP, IAPP, cofillin, and others (Wang et al., 2024). The findings reported here provide the first demonstration in the mammalian brain of a functional link between *Snca* and *Atp7a*, with a link to sex-specific effects of *Snca* function, as well. Thus, *Snca* function is protective in the context of the *Atp^I610^* mutation reported here.

Potential mechanisms of protective effects of *Snca* should be further examined in the context of the *Atp^I610S^* mutation. We suggest that lack of *Snca* may impact localization of *Atp7a^I610S^* by affecting its transport from the ER to the Golgi compartment, thus preventing its further transport to the trans-Golgi via possible mechanisms include two trans-Golgi mechanisms: inefficient SNARE assembly impeding vesicular transport between the two compartments or *Snca* activity as a chaperone to stabilize mutant *Atp7a* and facilitate trafficking to the trans-Golgi. Other interactions may occur in other cellular compartments, as well, including at the cell membrane where copper transport may be facilitated by *Atp7a* protein. One of the limitations of this study is the lack of data on whether the relationship between *Snca* and *Atp7a* proteins is direct or indirect; to date, there are no studies indicating a direct interaction between these two proteins and this manuscript reports the only evidence. Moreover, we observed, during the course of this study that the fertility of mice carrying the Atp7aI610S mutation is greatly reduced, thus making some studies more difficult. Nonetheless, further studies of primary cortical neurons from *Atp7a* mutant male mice may enable further localization studies and interaction studies with the mutant protein. A second limitation of this study is the fact that ENU mutagenesis screens in general have limitations in that the identification of interacting genes is skewed towards those that have the most-noticeable phenotypes, the longest DNA sequences and are viable to birth (Barbaric et al., 2007).

It is notable that Menkes disease has a strong neurological component, as does Parkinson’s disease. The loss of neuronal sub-types, that may lead to childhood or adult motor neuron disease, is not well understood. However, this work, and other recent studies, suggest that there are shared etiologies and molecular mechanisms underlying diverse neurodegenerative disorders. Multiple neurodegenerative disorders may be linked to deficits in the intricate system of copper transporters, exporters, copper chaperones and copper trafficking proteins. Dysregulated copper metabolism may result in diseases such as Menkes disease and Wilson disease (a disease of excess copper), as well as diseases linked to interacting genes (such as *SNCA*) or to dysfunction of metalloenzymes such as SOD1 which is linked to familial amyotrophic lateral sclerosis (ALS) (Gale and Aizenman, 2024; Mielke et al., 2024). Further examination of the interactions of *Atp7a* and *Snca* in Parkinson’s disease, as well as other neurodegenerative diseases, is merited.

## Methods

### ENU mutagenesis

C57BL/6J males were treated with ENU (Justice et al., 1999), allowed to recover fertility, and mated to *Snca* null females on a 129S6 background (Cabin et al., 2002). G1 males were used in further breeding to uncover sensitized recessive mutations. The *Atp7a* mutation described here arose in 2 G1 females of the same pedigree. Further backcrosses to produce G2 and G3 generations were to 129S6 males that were *Snca* null or wild type. *Snca* genotyping primers have been described (Cabin et al., 2002). All procedures on mice have been approved by the McLaughlin Research Institute IACUC, and the Institute’s Animal Resource Center is AAALAC accredited. Statistical analyses were performed with the StatView statistics package (SAS Institute, Cary, NC).

### Sequencing

Primers for sequencing the coding sequence of *Atp7a* (23 amplicons, 22 coding sequence exons) were chosen using the UCSC Genome browser^1^ link from mouse *Atp7a* to ExonPrimer^2^, with default settings. Sequencing was performed at the UC Davis Sequencing facility.^3^ Exon 7 primers used for assessing the I610S mutation are F: TAAGGCAATCCTGTGCTACG and R: TGATTCCAGAAGGTGGTTGAC, with an amplicon size of 162 bp.

### Western blots

Western blots were performed using whole brain lysates as described (Cabin et al., 2002). The primary antibodies used were chicken anti-*Atp7a* (Sigma), mouse anti-*Gapdh* (Millipore), and rabbit anti-neuron-specific enolase (Polysciences). Secondary hydrogen peroxidase-conjugated antibodies, goat-anti-chicken, goat-anti-mouse, and goat-anti-rabbit were obtained from GE HealthCare/Amersham, as was ECL Plus for chemiluminescent detection.

### Immunohistochemistry and immunofluorescence

Brains perfused with 4% formaldehye were dehydrated, embedded in paraffin, and cut in 10um sections. Immunohistochemistry was performed as described (Cabin et al., 2005), using the primary antibodies rabbit anti-cleaved caspase-3 (Trevigen) and chicken anti-*Atp7a* (Sigma), Meyer’s haematoxylin was obtained from Sigma. Biotinylated secondary antibodies and VectaStain were from Vector Laboratories. Immunofluorescence was performed as described (Cabin et al., 2005). Primary antibodies were chicken anti-*Atp7a* as above and rabbit anti-*TGOLN2* (AbCam). Secondary antibodies were Alexa-488 anti-rabbit (Invitrogen) and DyLight-549 anti-chicken (Jackson ImmunoResearch). SH-SY5Y cells were obtained from ATTC.

### Microscopy

Bright-field and immunofluorescence images were captured using a Zeiss Axio-Imager microscope; confocal microscopy was performed on an Olympus FV-1000.

## Data Availability

The datasets presented in this study can be found in online repositories. The names of the repository/repositories and accession number(s) can be found in the article/supplementary material. We have included all of the data in this manuscript; any other data is freely available by contacting the corresponding author or by accessing appropriate databases as indicated.

## Footnotes

^1^ http://genome.ucsc.edu

^2^ http://ihg.helmholtz-muenchen.ed/cgi-bin/primer/ExonPrimerUCSC

^3^ http://www.davissequencing.com/
